# Type I Collagen from the Skin of Barracuda (*Sphyraena* sp.) Prepared with Different Organic Acids: Biochemical, Microstructural and Functional Properties

**DOI:** 10.3390/jfb14020087

**Published:** 2023-02-03

**Authors:** Nur Nadiah Matarsim, Abdul Aziz Jaziri, Rossita Shapawi, Ruzaidi Azli Mohd Mokhtar, Wan Norhana Md. Noordin, Nurul Huda

**Affiliations:** 1 Faculty of Food Science and Nutrition, Universiti Malaysia Sabah, Kota Kinabalu 88400, Malaysia; 2 Faculty of Fisheries and Marine Science, Universitas Brawijaya, Malang 65145, Indonesia; 3 Borneo Marine Research Institute, Universiti Malaysia Sabah, Kota Kinabalu 88400, Malaysia; 4 Biotechnology Research Institute, Universiti Malaysia Sabah, Kota Kinabalu 88400, Malaysia; 5 Fisheries Research Institute, Batu Maung, Penang 11960, Malaysia; 6 Faculty of Sustainable Agriculture, Universiti Malaysia Sabah, Sandakan 90509, Malaysia

**Keywords:** barracuda skin, organic acids, acid extraction, characterization

## Abstract

This study was carried out to compare the extractability and characteristics of barracuda (*Sphyraena* sp.) skin collagen using various organic acids. Acetic-solubilized collagen (ASBS), lactic-solubilized collagen (LSBS) and citric-solubilized collagen (CSBS) yielded 6.77 g/100 g, 10.06 g/100 g and 8.35 g/100 g, respectively, and those yields were significantly different (*p* < 0.05). All acid-solubilized collagens were considered as type I because of their two alpha chains (α1 and α2) detected in acrylamide gel after electrophoresis. Ultraviolet–visible (UV–vis) analysis confirmed that ASBS, LSBS and CSBS had similar absorption peaks (230.5 nm) and the results were in accordance with other fish collagens. Under infrared (IR) and X-ray diffraction (XRD) analysis, the triple helical structure of type I collagens extracted from barracuda skin was maintained. From a thermostability study, all type I collagens showed a higher maximum transition temperature (*T_max_* = 40.16 to 41.29 °C) compared to other fish skin collagens. In addition, the functional properties of the extracted collagens revealed the ASBS had higher water and oil absorption capacities than the CSBS and LSBS samples. The highest level of the emulsion ability index (EAI) (>200 m^2^/g) was detected under acidic conditions (pH 4), while lower EAIs were recorded under the alkaline (pH 10) and neutral treatments (pH 7). All type I collagens had a higher relative solubility (>60%) at a low pH test but the solubility level sharply decreased at a neutral pH. In addition to this, a lower concentration of NaCl (0–20 g/L) showed the higher percentage of solubility (>60%) while adding over 30 g/L of NaCl decreased solubility (>40%). From a microstructural test, all type I samples had an irregular and dense flake structure with random coiled filaments. Overall, collagen extracted from the barracuda skin may be applied as an alternative collagen from an industry perspective.

## 1. Introduction

Barracuda (*Sphyraena* sp.), also known by its local name of Alu alu, belongs to the family Sphyraenidae. As a commercially important tropical marine species in Malaysia, its total production was approximately 7895 metric tons in the period of 2016 to 2019 [1]. This fish is often found near the water surface, with a silvery body and many dark bars running across its upper half. The caudal fin is generally black with white tips and juveniles have a series of large dark spots on the sides of their bodies [2]. Due to high production, barracuda is extensively used for diversified fish processing, such as dried and salted fish, fillet and surimi, as well as in surimi-based products (i.e., meatball, dumpling and nugget) [3]. During fish processing, however, a significant amount of fish byproducts is produced, including fish heads, viscera, fins, scales and skin, estimated at around 60 percent, and subsequently brings about negative impacts on the environmental and financial sectors. In terms of the environment, the underutilization of fish byproducts is wasted in aquatic and/or landfill areas, resulting in ecosystem destruction. In addition to this, the byproducts from fish processing are rich in organic acids and classified as a certified waste, requiring treatment before disposal, and this practice could increase the costs of the fish processing industry [4]. Hence, an effective approach in dealing with these issues is necessary, considering the high nutritional content derived from fish byproducts [5]. Collagen, a fibrous protein, is present in all multicellular organisms. It is an important structural protein that is responsible for the strength of tendons, skin and internal organs. Typically, collagen is governed by unique repeating sequences of glycine (G), proline (P) and hydroxyproline (O) in polypeptide chains. So far, more than 29 types of collagens have been recognized and each type is distinguished by its protein structure, amino acid motif and function [6,7]. Type I is the most abundant collagen (approximately 90 percent) found in the connective tissue of animals, including fish, cattle, pigs and poultry [8]. Moreover, type I collagen is extensively used in foods, cosmetics, pharmacies and biomedicines [9].

In some cases, collagens obtained from land-based animals could raise anxiety in end users because of certain transmissible diseases, such as foot-and-mouth disease (FMD), bovine spongiform encephalopathy (BSE) and avian flu (H5N1) [10]. Another reason is that some religious believers such as Muslims and Jews cannot consume porcine-based products, while Hindus do not eat cow and its derivatives [11]. As a choice, fish collagen has gained increasing attention in recent years. It is not only abundant in terms of its raw materials and safer in terms of infectious diseases, but it also has good biochemical properties after being modified [12]. Numerous research relating to fish collagen extraction processes from different species of fish has been documented. This includes research on bigeye snapper (*Priacanthus tayenus*) skin [13], lizardfish (*Saurida tumbil*) skin, bones and scales [14,15,16], parrotfish (*Chlorurus sordidus*) skin [17], sturgeon fish (*Huso huso*) skin [18], bigeye tuna (*Thunnus obesus*) skin, bones and scales [19], red stingray (*Dasyatis akajei*) skin [20], Siberian sturgeon (*Acipenser baerii*) cartilage [21], loach (*Misgurnus anguillicaudatus*) skin [22], tilapia (*Oreochromis niloticus*) scales [23], silver catfish (*Pangasius* sp.) skin [24], grey mullet (*Mugil cephalus*) scales [25], golden pompano (*Trachinotus blochii*) skin and bones [26], and grass carp (*Ctenopharyngodon idella*) skin, bones and scales [27]. Their characteristics have also been well evaluated. From those studies, collagens extracted from the skin of fish could be a promising alternative due to a higher yield compared to bone, cartilage and bone parts. In addition, fish skin collagen may be more prospective in developing new collagen-based products, particularly from an industrial viewpoint.

The extractability of fish collagen can be enhanced by adding different acid solutions. Using organic acids is more preferable than inorganic acids (hydrochloric acid/HCl). Our previous study reported that lizardfish prepared with the aid of organic acids had high yields and its properties were comparable to other fish collagens. The use of organic acids in producing barracuda skin collagen could provide basic information for further study. Therefore, this work aims to compare the extractability of barracuda skin collagen assisted by adding different organic acids (i.e., acetic, lactic and citric acids), and their biochemical, structural and functional characteristics are also discussed.

## 2. Materials and Methods

### 2.1. Materials

Barracuda fish (*Sphyraena* sp.) were supplied from a seafood market in Kota Kinabalu, Malaysia. Before delivery to the lab, thirty kilograms of ice were added to the fish (15 kg) in an ice box to maintain their freshness. Upon arrival, the barracuda were washed and prepared for skin separation using a separator machine (SFD-8, Taiwan). The separated skins were cut into small sizes (1.0 × 1.0 cm^2^) with a stainless steel knife, packed in a plastic container and subsequently stored in a freezer for further experimentation. Acrylamide, Coomassie Blue R-250, *N*,*N*,*N*′,*N*′-tetramethyl ethylene diamine (TEMED), sodium dodecyl sulphate (SDS), Folin–Ciocalteu’s phenol and acetic acid (glacial) were purchased from Merck (Darmstadt, Germany). Lactic acid solution (C_3_H_6_O_3_) was purchased from Bendosen (Selangor, Malaysia) and citric acid (C_6_H_8_O_7_) was bought from Systerm (Selangor Darul Ehsan, Malaysia). Tris-HCL, bovine serum albumin and Lowry reagent were supplied from Sigma-Aldrich (St. Louis, MO, USA). Precision Plus Protein Dual Color standards (markers) were purchased from Bio-Rad Laboratories (Hercules, CA, USA). Other chemicals used in this experiment were of analytical grade.

### 2.2. Preparation of Acid-Solubilized Collagen (ASC)

**Figure 1:**
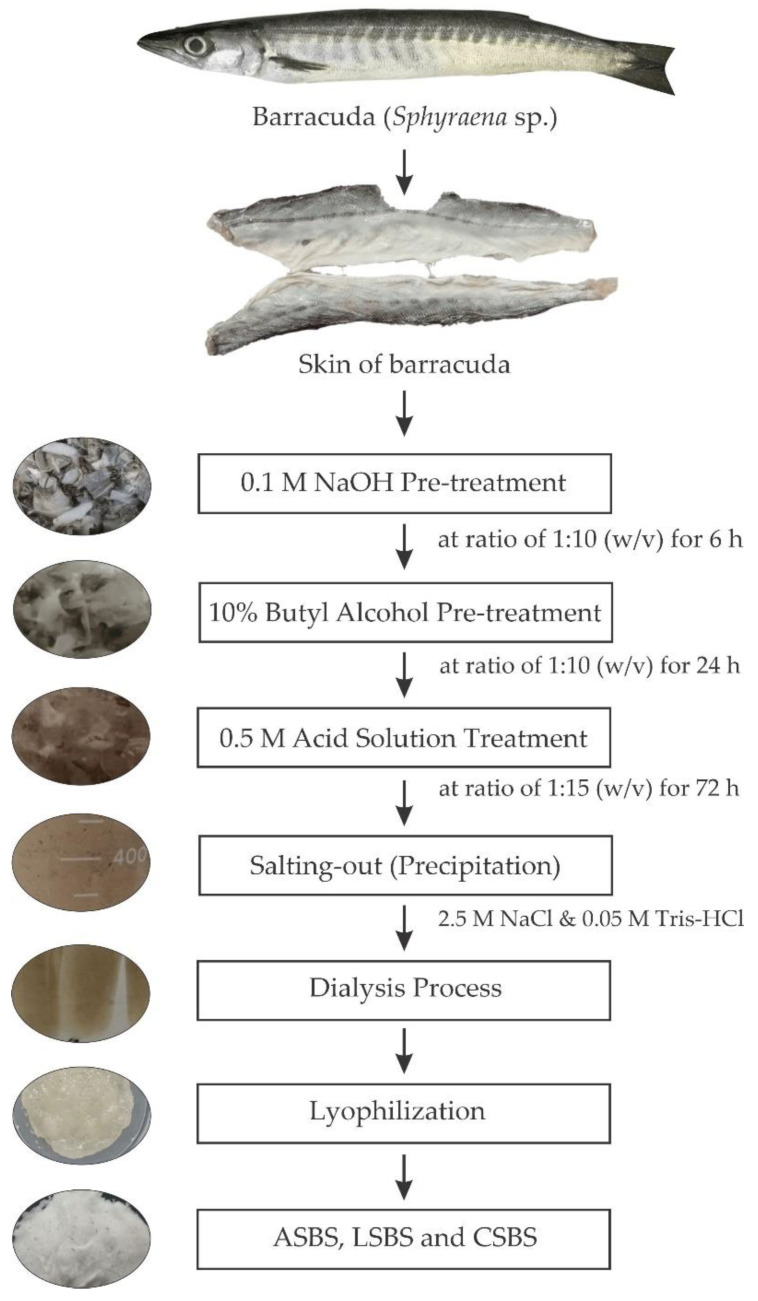
Flowchart of acid-solubilized barracuda skin collagen production. ASBS: acetic acid-solubilized barracuda skin collagen; LSBS: lactic acid-solubilized barracuda skin collagen; CSBS: citric acid-solubilized barracuda skin collagen.

The acid solubilization of the barracuda (*Sphyraena* sp.) skin was conducted according to the procedures by Jongjareonrak et al. [28] and Jaziri et al. [14], with minor amendments. The process flow of each extraction step is illustrated in Figure 1, and all procedures were carried out in a cold lab (4 °C). Fifty kilograms of barracuda skin were immersed in a solution of sodium hydroxide (NaOH, 0.1 M) at a ratio of 1:10 (*w*/*v*) for 6 h with continuous stirring. The alkaline solution was changed every 3 h and the samples were neutralized with distilled water. The first pre-treatment comprised the removal of non-collagenous protein and pigmentation. The neutralized samples were then pretreated with 10% butyl alcohol at a ratio of 1:10 (*w*/*v*) for 24 h with the changing of the solution twice to eliminate fat from the samples. The pretreated samples were rinsed with cooled distilled water for 30 min, replacing every 10 min. After the pre-treatment process, the samples were further solubilized with acetic, lactic and citric acids at a ratio of 1:15 (*w*/*v*) for 72 h. The acid-solubilized samples were then filtered through a double layer of cheesecloth. The filtrates were subsequently precipitated with 2.5 M of sodium chloride (NaCl) and 0.05 M Tris-HCl at a pH 7.0. Next, the precipitates were adjusted to centrifugation at 15,000× *g* rcf for 15 min. After that, acid solutions (0.5 M) were used to dissolve the pellets at a ratio of 1:1 (*w*/*v*). The solutions were further allowed to dialyze in 15 volumes of acid solutions (0.1 M) for 1 day, followed by distilled water for 2 days. Finally, the dialysates were lyophilized using a freeze dryer (Labconco, Kansas City, MO, USA) and then stored in a freezer until the next experimentation. The dried collagens were named acetic acid-solubilized barracuda skin collagen (ASBS), lactic acid-solubilized barracuda skin collagen (LSBS) and citric acid-solubilized barracuda skin collagen (CSBS).

### 2.3. Analyses

#### 2.3.1. Determination of Yield and Hydroxyproline (Hyp) Composition

The yields of the acid-solubilized collagens (ASBS, LSBS and CSBS) were determined according to the method described by Jongjareonrak et al. [28] as mentioned below:

(1) $$\mathrm{Yield}\left(\%\right)=\frac{\mathrm{Weight}\;\mathrm{of}\;\mathrm{lyophilized}\;\mathrm{collagen}}{\mathrm{Weight}\;\mathrm{of}\;\mathrm{initial}\;\mathrm{wet}\;\mathrm{barracuda}\;\mathrm{skin}}\times 100$$

The Hyp content of the solubilized collagens was retrieved based on the protocol by Bergman and Loxley [29]. The ASBS, LSBS and SCBS samples were hydrolyzed with an acid solution (6 M HCl) at 110 °C for 1 day. After hydrolysis, the ASC hydrolysates were subjected to filtration through a No. 4 Whatman filter paper and subsequently adjusted at a pH 6.5–7.0. About 200 µL of hydrolysates were put into test tubes (15 mL) and then 400 µL isopropyl alcohol was added. Next, 200 µL of oxidant solution (freshly prepared) was added into the tubes and they were stood for 5 min at room temperature. Ehrlich’s reagent solution (2.3 mL) was further added and thoroughly mixed. Then, the mixed samples were incubated for 25 min at 60 °C. The incubated samples were subsequently cooled at room temperature and up to 10 mL was combined with a 2-propanol solution. Absorbances against distilled water were measured using a spectrophotometer at a wavelength of 558 nm. Hydroxyproline standard solution (10 to 70 ppm) was also prepared in this study.

#### 2.3.2. Color Attributes

The color parameters of the ASBS, LSBS and CSBS samples from the barracuda skin were obtained using the established procedure [30]. All samples were tested under a colorimeter (ColorFlex CX2379; HunterLab, Reston, VA, USA). The attributes designed in the present study were L*, representing the lightness value; a*, reflecting the redness value; and b*, depicting the yellowness value.

#### 2.3.3. Sodium Dodecyl Sulfate-Polyacrylamide Gel Electrophoresis (SDS-PAGE)

All samples (ASBS, LSBS and CSBS) were tested under SDS-PAGE to obtain their molecular weight and collagen type. The method used in this study referred to the previous research method from Laemmli [31] with minor adjustments. Around 2.5 mg of lyophilized collagens were dissolved in a 5% SDS solution and thoroughly mixed. Next, the mixed samples were treated at a high temperature (85 °C) for 60 min and afterward subjected to centrifugation at 8500× *g* rcf for 5 min to remove undissolved matter. After that, fifteen microliters of supernatants was mixed equally with the sample buffer (50 mM Tris-HCl, pH 6.8; 2% SDS; 12.5 mM EDTA; 10% glycerol; and 0.02% bromophenol blue), treated with and without 10% β-mercaptoethanol (β-ME). The mixtures were heated at the same temperature for 5 min and subsequently loaded onto a polyacrylamide gel (4% stacking gel and 7.5% resolving gel). The gel was electrophoresed with a constant voltage of 120 volts for about 70 min. After electrophoresis, the gel was subjected to staining for approximately 30 min. Furthermore, the stained acrylamide gel was destained by adding prepared destaining solution, containing 10% (*v/v*) acetic acid and 30% (*v/v*) methanol solutions. The electrophoretic bands that appeared were comparable to the protein marker (precision plus protein dual color standards).

#### 2.3.4. Attenuated Total Reflectance–Fourier Transform Infrared Spectroscopy (ATR–FTIR)

IR spectra of the acid-solubilized collagens from the barracuda skin were analyzed using an FTIR spectrometer instrument (Cary 630; Agilent, Santa Clara, CA, USA). The procedure employed in the present work was prepared from the study by Matmaroh et al. [32]. Ten milligrams of dried collagens were placed on the spectrometer’s crystal cell. A background spectrum made from clean, empty cells at an ambient temperature served as the basis for all the spectra, which were all designed with a resolution of 2 cm^−1^ throughout a wavenumber range of 4000–500 cm^−1^ for 32 scans. The spectrophotometer data were then read with the provided Agilent Microlab software tool.

#### 2.3.5. Ultraviolet–Visible (UV–vis) Spectra of Acid-Solubilized Collagens

UV–vis absorption spectra of the ASBS, LSBS and CSBS samples were obtained based on the protocol by Jaziri et al. [14], using a UV–vis spectrophotometer (Cary 60; Agilent, Santa Clara, CA, USA). Five milligrams of lyophilized barracuda skin collagens were prepared by dissolving them with acetic acid solution and the solutions were then vortexed until solubilized. Then, the mixtures were subjected to centrifugation at 8500× *g* for 15 min to separate the supernatants. The supernatants were further dropped into a quartz cell. The spectrophotometer wavelengths were set in a range of 350 nm to 200 nm with a baseline of acetic acid solution.

#### 2.3.6. X-ray Diffraction (XRD) Test

X-ray diffraction analysis of the ASBS, LSBS and CSBS samples was run under an X-ray diffraction instrument (Rigaku Smart Lab^®^, Japan). All procedures were adopted in accordance with the study by Chen et al. [20]. The freeze-dried barracuda skin collagens were examined using the XRD instrument and the X-ray source used during scanning was a copper Kα. The current and tube voltages were set at 50 mA and 40 kV, respectively. The scanning condition was prepared by measuring from 10° to 40° (2θ) with a speed of 0.06° per second.

#### 2.3.7. Thermostability Analysis

The dried barracuda skin collagens, i.e., ASBS, LSBS and CSBS, were subjected to thermostability evaluation, using a Perkin-Elmer DSC machine (Norwalk, CA, USA). All samples were dissolved by adding deionized water at a ratio of 1:40 (*w*/*v*) to rehydrate the collagens. Next, the rehydrates were stored in a chiller for 48 h. After rehydration, the prepared samples were precisely weighed into an aluminum pan and sealed tightly. Prior to analysis, an indium (5.55 mg) was used as a standard in calibrating the DSC instrument. After that, the sealed collagen samples were scanned, with temperatures initiated from 20 °C to 50 °C at a rate of 1 °C per minute. An empty pan was employed as a reference. After scanning, the maximum transition temperature (*T_max_*) and total denaturation enthalpy (Δ*H*) were determined by adjusting from the thermogram’s endothermic peak and area, respectively [32].

#### 2.3.8. Scanning Electron Microscopy (SEM)

The microstructural characteristics of the ASBS, LSBS and CSBS samples were scanned by scanning electron microscopy (Sigma; Zeiss, Germany). A gold sputter coater (JEOL JFC-1200; Tokyo Rikakikai Co., Ltd., Tokyo, Japan) was used to coat the barracuda skin collagens. Subsequently, the coated acid-solubilized collagens were captured with a magnification of 500× [33].

#### 2.3.9. Solubility Profile

Solubility tests at different pHs and NaCl concentrations were carried out according to a previous study [14,15,16]. For pH treatment, the samples were prepared by dissolution with acetic acid solution (0.5 M). Then, the solutions were adjusted at different pH conditions, with 2.5 M HCl and 2.5 M NaOH solutions used for the pH adjustment process. The treated collagens with various pH values were further stirred for 1 h and centrifuged at 8500× *g* rcf for 20 min. In terms of the NaCl treatment, about 5 mL of solubilized collagens were transferred into 5 mL of prepared NaCl concentrations (0–50 g/L). The NaCl-treated samples were then stirred continuously for 1 h in a chiller. After that, the collagen samples were centrifuged at 8500× *g* rcf for 20 min. All pH- and NaCl-treated collagens were determined by their protein content, using the established procedure by Lowry et al. [34]. A standard curve was prepared by dissolution in bovine serum albumin (BSA). The relative solubilities (%) of the barracuda skin collagens were measured using the following formula:

(2) $$\mathrm{Relative}\;\mathrm{solubility}\;\left(\%\right)=\frac{\mathrm{Current}\;\mathrm{concentration}\;\mathrm{of}\;\mathrm{protein}\;\mathrm{at}\;\mathrm{NaCl}\;\mathrm{or}\;\mathrm{pH}}{\mathrm{The}\;\mathrm{highest}\;\mathrm{concentration}\;\mathrm{of}\;\mathrm{protein}}\times 100$$

#### 2.3.10. Water Absorption Capacity (WAC) and Oil Absorption Capacity (OAC)

The WAC and OAC tests of all the barracuda skin collagens referred to the method by Chen et al. [20]. For the WAC test, fifty milligrams of dried collagen samples were immersed with deionized water (1 mL) and then centrifuged at 10,000× *g* rpm for 10 min. Then, the centrifuged samples were stood for 10 min at room temperature before being centrifuged again at 15,000× *g* rpm for 20 min. After that, the volumes of the supernatants were recorded. Meanwhile, the same procedure was also applied to determine the OAC, with sunflower oil being used to dissolve the dried collagens. Both the WAC and OAC were determined using the formula:

(3) $$\mathrm{WAC}\;\mathrm{or}\;\mathrm{OAC}\;(\mathrm{mL}/g)=\frac{V_{1}-V_{2}}{\mathrm{Weight}\;\mathrm{of}\;\mathrm{lyophilized}\;\mathrm{collagen}\;(g)}$$

where V_1_ represents the volume of water/oil dropped before centrifugation (mL) and V_2_ is the volume of water/oil after centrifugation (mL).

#### 2.3.11. Emulsion Ability Index (EAI)

The EAI was determined using the method developed by Chen et al. [20]. Dried collagen samples were first dissolved with distilled water (0.5% *w*/*v*). Next, about 6 mL of sample solutions were subjected to various pH adjustments (pH 4.0, pH 7.0 and pH 10.0). After pH adjustment, two milliliters of sunflower oil were directly added to the prepared sample solutions and then the samples were homogenized at 16,000 rpm for 2 min using a homogenizer (Ultra Turrax^®^; Atkinson, NH, Enfield, CT, USA). The homogenized solutions were taken up to 50 µL at 0 min and 10 min using a micropipette (Eppendorf, USA). A total of 5 mL of 0.1% SDS was then added to the solutions. The absorbances of each of the treated samples were measured at a wavelength of 500 nm using a UV–vis spectrophotometer and 0.1% SDS was used as a blank. The EAI was calculated based on the following equation where A0 is the absorbance at 0 min:

(4) $${\mathrm{EAI}\;(m}^{2}/g)=\frac{2\times 2.303\times A_{0}}{0.25\times \mathrm{collagen}\;\mathrm{weight}\;(g)}$$

### 2.4. Statistical Analysis

The experiments were performed in triplicate with a completely randomized design (CRD). Data were expressed as the means with standard deviations, and the significance level was marked by *p* < 0.05. To compare the means, one-way ANOVA and Duncan’s multiple range test were used in SPSS Statistics version 28.0. (IBM Corp., Armonk, New York, NY, USA).

## 3. Results and Discussion

### 3.1. Yield and Hyp Content

The yields of the ASBS, LSBS and CSBS samples are presented in Table 1. The obtained results showed significant effects (*p* < 0.05) on the yields of three different samples, with the collagen solubilized using a lactic acid solution (0.5 M) having the highest yield compared to the citric and acetic acid solutions. It has been suggested that the application of lactic acid during the solubilizing process could increase the extractability of collagen from barracuda skin. Comparably, other fish skin collagens extracted using acid to aid the procedure in pangasius catfish (*Pangasius* sp.) [24], sailfish (*Istiophorus platypterus*) [35] and sturgeon (*H. huso*) [18] yielded 5.47–10.94 g/100 g, 5.76 g/100 g and 9.98 g/100 g, respectively. However, our samples were of a lower yield than the other acid-solubilized collagens from the skins of lizardfish (*S. tumbil*) (11.73 g/100 g) [14], Spanish mackerel (*Scomberomorus niphonius*) (13.7 g/100 g) [36] and bigeye tuna (*Thunnus obesus*) (13.5 g/100 g) [19]. The difference in the yields of the aforementioned fish skin collagens could be due to the raw material of the fish species used. Moreover, chemicals and extraction processes might affect the yield of fish collagens [14]. In terms of Hyp composition, the ASBS sample exhibited higher Hyp content (82.78 ± 0.19 mg/mg) (*p* < 0.05) than in the LSBS (81.76 ± 0.05 mg/g) and CSBS (81.97 ± 0.14 mg/g) samples. These findings agreed with the hydroxyproline compositions from the various fish collagens such as lizardfish (*S. tumbil*) (80.94–83.29 mg/g) [16], tilapia (*Oreochromis mossambicus*) (76–80 mg/g) [23], bigeye tuna (*Thunnus obesus*) (82–87 mg/g) [19] and miiuy croaker (*Miichthys miiuy*) (85 mg/g) [37]. The Hyp (mg/g) content in various fish collagens could be influenced by many factors, including species, age, size, composition and structure found in the fish tissue, and the procedure of collagen extraction [38].

### 3.2. Color Attributes

**Figure 2:**
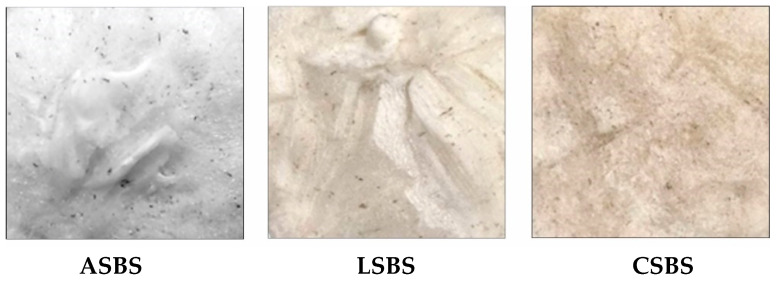
The appearance of barracuda skin collagens. ASBS: acetic acid-solubilized barracuda skin collagen; LSBS: lactic acid-solubilized barracuda skin collagen; CSBS: citric acid-solubilized barracuda skin collagen.

The color attributes of barracuda skin collagen prepared with various acids were also collected. Table 1 describes that all extracted collagens were significantly different (*p* < 0.05) in their L*, a* and b* values. Particularly, the ASBS sample had the lightest attributes compared to both the LSBS and CSBS samples (Figure 2), indicating that the acetic acid solution used in the study could effectively generate fish collagen with a high lightness parameter. Our present study (ASBS) also showed a greater L* value than previous research observed in lizardfish (*S. tumbil*) skin collagen (72.76) [14] and barramundi (*Lates calcarifer*) skin collagen (44.76–65.41) [39]. In contrast, snakehead fish (*Channa argus*) skin collagen prepared by adding a certain amount of hydrogen peroxide (H_2_O_2_) solution showed a lighter attribute (L* = 89.49) [40] compared to our data, suggesting that the use of H_2_O_2_ solution could provide a brighter collagen product. As stated by Sadowska et al. [41], collagen with a brighter color is more desirable for new product developments, such as foods, pharmaceuticals and medicals, because it has less or even no interference with the original color of the product. For the a* and b* values, the LSBS and CSBS samples, respectively, possessed the highest values. Therefore, if the L* value of a product is high, then the a* and b* values are low, and vice versa.

### 3.3. SDS-PAGE Profile

**Figure 3:**
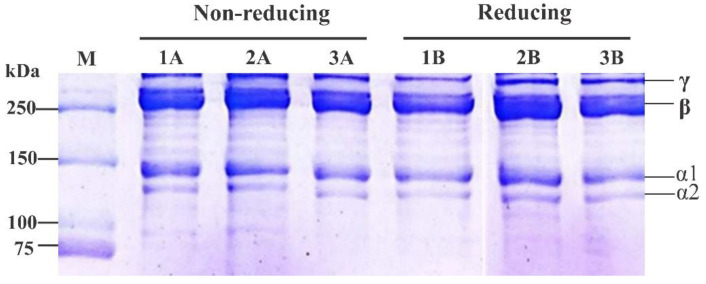
Electrophoretic patterns of acid-solubilized collagens from barracuda skin. M: protein marker; 1A and 1B: acetic acid-solubilized collagen (ASBS); 2A and 2B: lactic acid-solubilized collagen (LSBS); 3A and 3B: citric acid-solubilized collagen (CSBS).

Electrophoretic patterns from the ASBS, LSBS and CSBS samples are depicted in Figure 3. There were similar patterns of electrophoretic bands observed in all acid-solubilized collagens, representing two alpha chains (i.e., α1 and α2), one β- and one γ-chain. For molecular weight (MW) prediction, those alpha chains were at around 143.2 kDa and 136.6 kDa, respectively, with the band intensity of α1 almost 2-fold as high as α2. As reported in the study by Benjakul et al. [42], fish collagen composed of two alpha chains was categorized as type I collagen. This statement confirmed that the barracuda skin collagens prepared with different acids were type I collagen and the obtained results were comparable to previous documentation of fish collagens, including seabass (*L. calcarifer*) (α1 = 118 kDa and α2 = 105 kDa) [1], loach fish (*M. anguillicaudatus*) (α1 = 127 kDa and α2 = 115 kDa) [22], tilapia (O. *niloticus*) (α1 = 125 kDa and α2 = 114 kDa) [23] and golden pompano (*T. blochii*) (α1 = 120 kDa and α2 = 100 kDa) [26]. Furthermore, the β- and γ-chains observed in all acid-solubilized collagens were predicted, respectively, as 255.0 kDa and 322.8 kDa, representing dimer and trimer bands, as also investigated in our previous reports of fish collagens [14,15,16]. Under reducing (with β-ME) and non-reducing (no β-ME) treatment, there was no difference in the electrophoretic patterns of the ASBS, LSBS and CSBS samples, suggesting no disulfide (S–S) bonds found in all acid-solubilized collagens, as reported in the literature.

### 3.4. UV–vis Absorption

**Figure 4:**
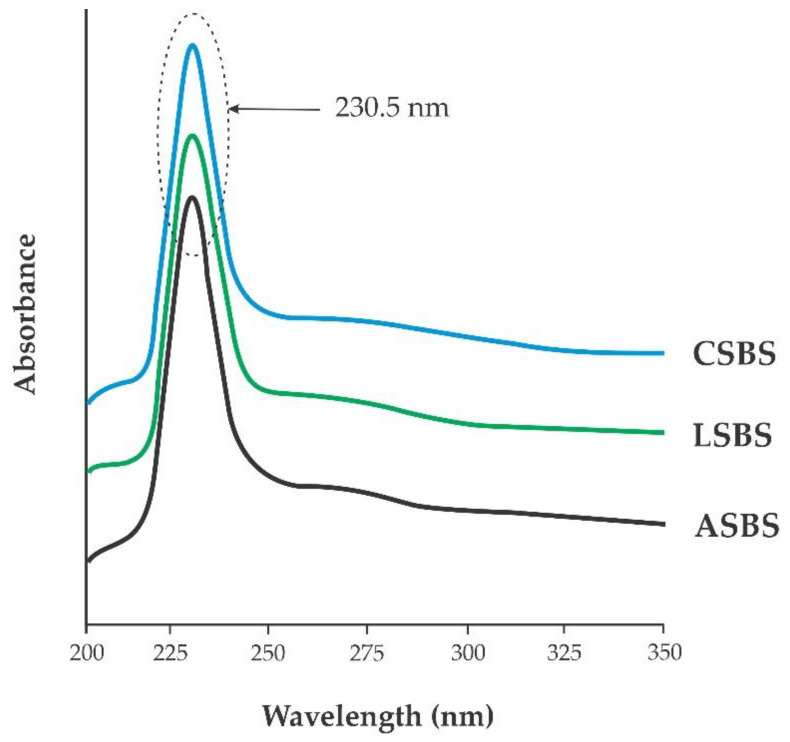
UV–vis spectra of acid-solubilized collagens from barracuda skin. ASBS: acetic acid-solubilized barracuda skin collagen; LSBS: lactic acid-solubilized barracuda skin collagen; CSBS: citric acid-solubilized barracuda skin collagen.

Generally, fish collagen possesses a significant peak absorption spectrum at about 210 nm to 240 nm [43]. Figure 4 confirms the maximum spectra of the ASBS, LSBS and CSBS were located at the same wavelength (230.5 nm). These detected spectra were closely associated with the functional groups of carboxyl (-COOH), carbonyl (C=O) and amide (CONH_2_), which represent the polypeptide chains of fish collagen, as reported by Jaziri et al. [14]. Besides those, at the wavelengths of 300–250 nm, all acid-solubilized collagens presented low absorption peaks. This was probably indicative of some aromatic amino acid (e.g., phenylalanine, tryptophan, tyrosine). The UV absorption spectra observed in our present study were similar to various fish collagens, such as in the lizardfish (*S. tumbil*) [16], red drum (*Sciaenops ocellatus*) [44], miiuy croaker (*M. miiuy*) [37], Siberian sturgeon (*A. baerii*) [21] and puffer fish (*Lagocephalus inermis*) [45].

### 3.5. Attenuated Total Reflection–Fourier Transform Infrared Spectroscopy (ATR–FTIR)

**Figure 5:**
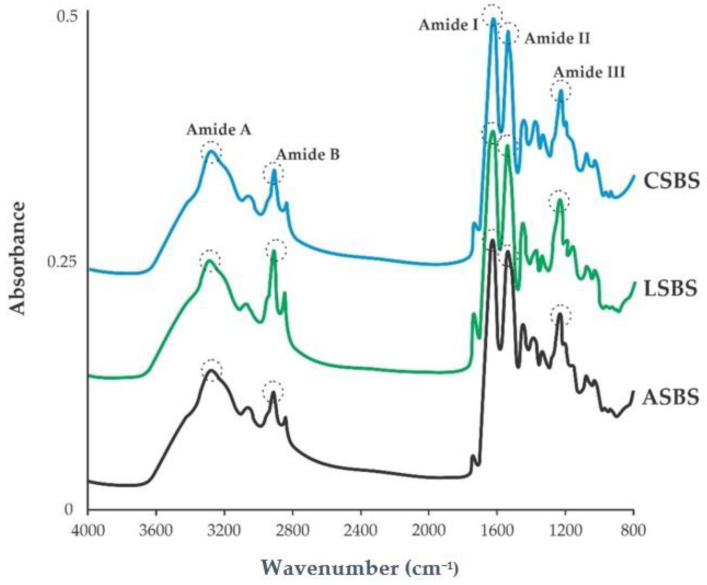
IR spectra of acid-solubilized collagens from barracuda skin. ASBS: acetic acid-solubilized barracuda skin collagen; LSBS: lactic acid-solubilized barracuda skin collagen; CSBS: citric acid-solubilized barracuda skin collagen.

Figure 5 presents the IR spectra of acid-solubilized collagen from the skin of barracuda, and the peak locations and their descriptions are also provided in Table 2. All samples used in this study had various prominent peaks, such as Amide I–III, Amide A and Amide B. These peak areas were also observed in other fish collagens, such as in the Nile tilapia (*O. niloticus*) [23], lizardfish (*S. tumbil*) [14], purple-spotted bigeye snapper (*P. tayenus*) [13], sturgeon fish (*H. huso*) [18], barramundi (*L. calcarifer*) [11] and loach (*M. anguillicaudatus*) [22]. To assess the stability of the triple helical structure of fish collagen Benjakul et al. [42] proposed the formula of Δ*v(v_I_-v_II_)*, where the difference in wavenumber (cm^−1^) between Amides I and II resulted in less than 100 cm^−1^, indicating the triple helical structure of extracted collagen was preserved [46]. Our data presented that the triple helical structures of the ASBS, LSBS and SCBS samples were preserved, suggesting that the use of acidic solution during the extraction process could solubilize the collagens without damaging the collagen structures. Moreover, another way of testing the structure of collagen’s triple helix is by dividing the value between Amide III and the 1450 cm^−1^ bands (AIII/A1450), as published by a previous study [47]. The obtained results expressed that the triple helical structures of the ASBS, LSBS and CSBS samples from the barracuda skins did not change during the extraction process as described from their absorption ratio values (~1.0).

### 3.6. Evaluation of X-ray Diffraction (XRD)

The XRD data of the ASBS, LSBS and CSBS are described in Table 3. There were two prominent peaks detected in all acid-solubilized collagens, with the first peak (peak 1 = 7.26–7.64°) depicting a sharp form and the second peak (peak 2 = 19.12–20.02°) reflecting a broad form. The obtained XRD peaks in the present study were generally portrayed as triple helical structures of collagen, as characterized in the collagen standard derived from the skin of a calf sample [16]. Other researchers investigating various fish collagens have also reported the same diffraction patterns in fish collagens, including, for instance, in lizardfish (*S. tumbil*) skin, scales and bone collagens [14,15,16], golden pompano (*T. blochii*) skin and bone collagens [26], carp fish (*Cyprinus carpio*) scale collagen [48] and three genetic lines of tilapia (*O. niloticus*) skin collagen [49]. Moreover, d values were used to estimate the minimum values of repeated spacing by applying Bragg’s formula [48]. As tabulated in Table 3, the data in the first peak (d = 1.16–1.22 nm) represent the distance between the molecular chains of the triple helical structure of fish collagen and the d values of all the barracuda skin collagens were almost all the same. For the second peak, the d values of ASBS, LSBS and CSBS were between 0.44 nm and 0.46 nm (Table 3), and thus relatively similar to each other. This peak depicted the distance between the skeletons of the collagen structure. The diameter (d) of a collagen molecule with a single left-handed helix chain and a triple helix structure matched the diameters of the collagens from the barracuda skin prepared by solubilizing them with different acids. Overall, our extracted collagens showed no denaturation in the structures of their triple helices and were in their native conformations.

### 3.7. Thermal Stability Evaluation

Using differential scanning colorimetry (DSC), the thermal stability measured with a *T_max_* value for all collagens (ASBS, LSBS and CSBS) was evaluated. The ASBS had the highest *T_max_* value (41.29 °C) compared to the LSBS (40.69 °C) and CSBS (40.16 °C) (Table 3). A higher *T_max_* value observed in the barracuda skin collagens described a higher thermal stability, suggesting all collagen samples used in this study had high hydroxyproline content, as noted in Table 1. The thermal stability of all samples was probably influenced by their imino acid composition, especially at the pyrrolidine rings that were partially constructed by H-bonding via the hydroxyl group of hydroxyproline [35]. Additionally, hydroxyproline could stabilize the structure of the triple helix through H-bonding in the coil-coiled α chains [50]. Much of the literature has informed that different fish collagens, such as in lizardfish (*S. tumbil*) skin (*T_max_* = 40.24 °C) [14], seabass (*L. calcarifer*) skin (*T_max_* = 39.32 °C) [11], loach (*M. anguillicaudatus*) skin (*T_max_* = 36.03 °C) [22], rohu (*L. rohita*) (*T_max_* = 36.40 °C) [51] and grass carp (*C. idella*) (*T_max_* = 35.60 °C) [27] have high thermostability due to being extracted from tropical fish species, while tempered fish species such as Spanish mackerel (*S. niphonius*) show lower *T_max_* values (14.66–16.85 °C) [36]. The Δ*H* value is the narrow area under the thermogram peaks, reflecting a number of energies used to uncouple the alpha chains of collagen and then turn them into random coils. The results confirmed the Δ*H* value of the ASBS sample was greater than those of LSBS and CSBS, implying a higher energy used in ASBS compared to the other samples. However, variation in the *T_max_* and Δ*H* values of collagen extracted from fish byproducts depends on the imino acid content, extraction process, fish species and other external factors, and particularly, habitat and water temperature [52].

### 3.8. Microstructure Analysis

**Figure 6:**
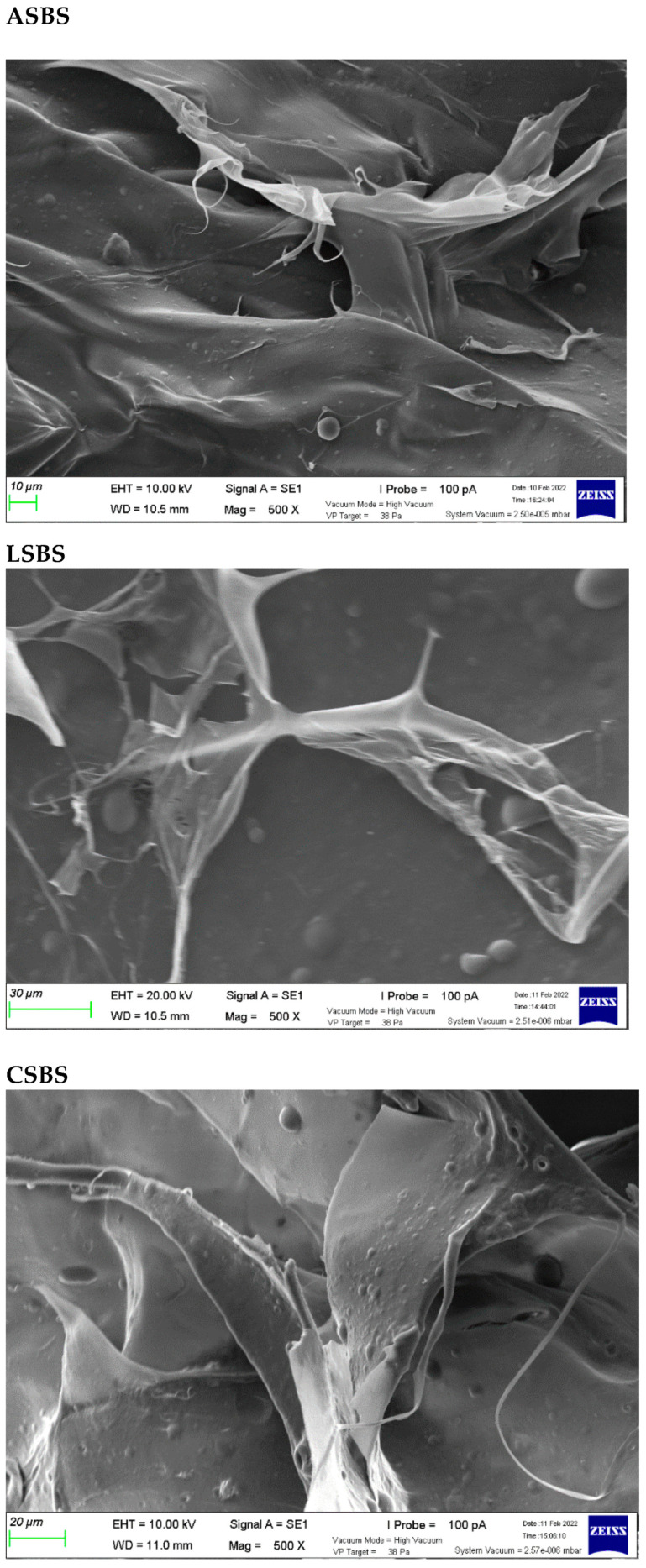
Scanning electron microscopy photographs of acid-solubilized collagens from barracuda skin. ASBS: acetic acid-solubilized barracuda skin collagen; LSBS: lactic acid-solubilized barracuda skin collagen; CSBS: citric acid-solubilized barracuda skin collagen.

Figure 6 depicts the morphological structure of ASBS, LSBS and CSBS portrayed by scanning electron microscopy (SEM). All samples presented a multi-layered form with the irregular sheet-like film connected by random-coiled filaments. The fibril-forming structures were also visible in all acid-solubilized collagens from the barracuda skin. Additionally, at a magnification of 500×, the porous and wrinkled structures were also observed in this study. This finding could be due to the dehydration during the lyophilization process as reported in the study by Schuetz et al. [53]. Some of the literature working on different fish collagens also supported our present work, such as on black ruff (*Centrolophus niger*) skin [54], lizardfish (*S. tumbil*) skin, bones and scales [14,15,16], marine eel (*Evenchelys macrura*) skin [55] and miiuy croaker (*M. miiuy*) scales [37]. Moreover, Lim et al. [9] stated that fish collagen products with fibrillary, interconnected and sheet-like film structures might be a promising source of material for nutraceutical, pharmaceutical and biomedical applications (e.g., bone and skin tissue development, wound dressing, cell migration and film coating components).

### 3.9. Solubility Profiles

**Figure 7:**
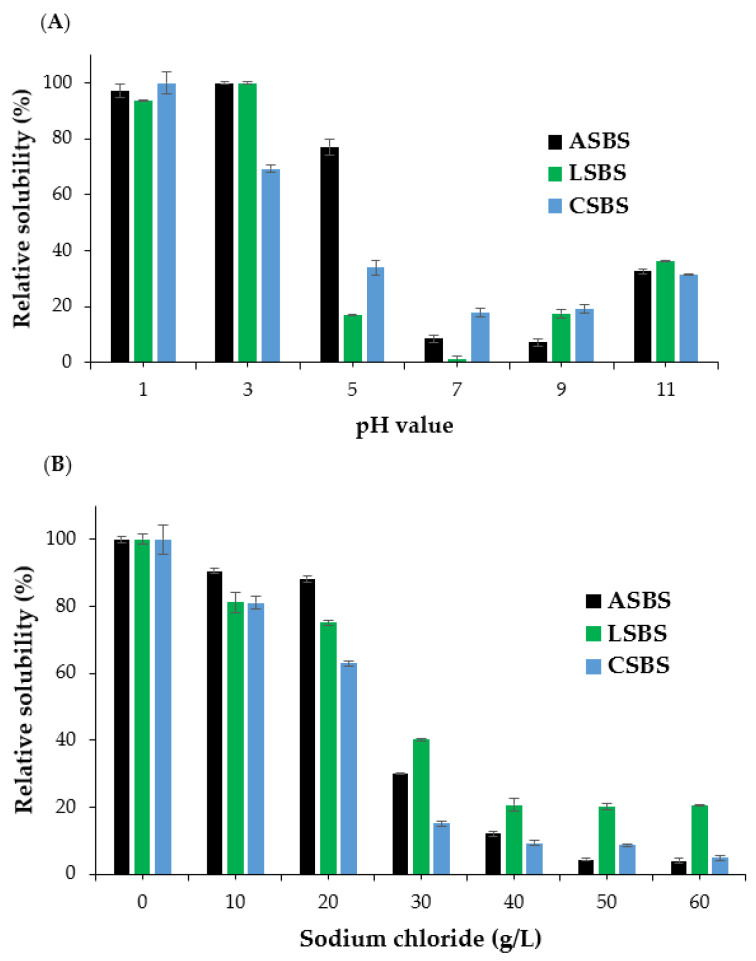
Relative solubility of acid-solubilized collagens from barracuda skin at (**A**) different pH levels and (**B**) sodium chloride concentrations. ASBS: acetic acid-solubilized barracuda skin collagen; LSBS: lactic acid-solubilized barracuda skin collagen; CSBS: citric acid-solubilized barracuda skin collagen.

Figure 7 shows the solubility profiles of ASBS, LSBS and CSBS treated under different pH levels and sodium chloride (NaCl) concentrations. All samples had a high solubility (>60%) under strong acidic conditions (pH 1.0 and pH 3.0), with the highest solubilization observed at pH 3.0 for ASBS and LSBS, while the CSBS sample was detected at pH 1.0 (Figure 6A). Another finding was that at pH 5.0, the barracuda skin collagen solubilized with acetic acid still had a high solubility (near 80%), indicating the acetic acid solution could effectively solubilize barracuda skin collagen under acidic conditions (ranging from pH 1.0 to pH 5.0). Nevertheless, at a neutral pH (pH 7.0) and in alkaline solution (pH 9.0 and pH 11.0), the relative solubility (RS) of the ASBS, LSBS and CSBS decreased sharply (<40%), with the lowest solubility found at pH 7.0. From these results, it could be assumed that collagen molecules show an increase in hydrophobic–hydrophobic interactions and subsequently, the total net charge of the protein becomes zero, particularly at the isoelectric point (pI), which is commonly found at a nearly acidic and neutral pH [28]. Basically, our experiments were comparable to the previous studies on various fish collagens, including on the purple-spotted bigeye snapper (*P. tayenus*) [13], tilapia (*O. niloticus*) [23], horse mackerel (*Trachurus japonicus*) [25], grey mullet (*M. cephalus*) [25] and lizardfish (*S. tumbil*) [14]. In terms of the different NaCl treatments, a higher solubility (>60%) in the collagens extracted from the skin of barracuda was recoded at low concentrations (0–20 g/L) of sodium chloride (Figure 7B). However, the RS (%) decreased intensely when NaCl was added gradually. The reason for this might be due to the high concentration of salt added during the salting out process. Chen et al. [44] found that protein precipitation was caused by an increase in salt concentration, hydrophobic–hydrophobic interactions within collagen polypeptide chains and competition for water. Our results were in agreement with the collagens isolated from the Spanish mackerel (*S. niphonius*) [36], lizardfish (*S. tumbil*) [14], golden pompano (*T. blochii*) [26] and Siberian sturgeon (*A. baerii*) [21].

### 3.10. Water/Oil Absorption Capacities

The water and oil absorption capacities (WACs and OACs, respectively) for the collagens from barracuda skin are presented in Table 4. There was no significant difference (*p* > 0.05) in the WAC found in the ASBS, LSBS and CSBS samples; however, ASBS showed a higher WAC value than the others. Meanwhile, for the OAC test, ASBS and CSBS were higher compared to the LSBS sample, with a significant difference (*p* < 0.05). The WACs and OACs of acid-solubilized barracuda skin collagens had greater values than those observed in glutelin [56], but our current study had lower WAC and OAC values in comparison with other fish collagens, e.g., red stingray (*D. akaje*) skin [20] and Nile tilapia (*O. niloticus*) skin [57]. In general, OAC is closely associated with non-polar amino acid groups, while WAC is related to hydrophilic groups. The hydrophobic interaction occurring between the non-polar amino acids of a collagen molecule and oil hydrocarbon chains could determine the OAC value of a collagen product [20]. In addition, the higher WAC values in the barracuda skin collagens could suggest that the acid-solubilized collagens used in this study had a higher hydrophilic group of amino acids, but lower group of non-polar amino acids.

### 3.11. Emulsion Property

Emulsion is an important characteristic of protein-based products. It is defined as an interface of water with oil (water–oil interface), and the EAI is used to determine the amount of oil that can be emulsified per unit of protein [37]. The EAI value for all barracuda skin collagens was significantly different (*p* < 0.05). A low EAI level was found under neutral conditions (pH 7.0). This might be due to the generally low solubility of acid-solubilized collagens when the pH is near the isoelectric point (pI). In addition, this could be due to changes in the electrostatic charge on the protein molecules and a decrease in the repulsive forces, resulting in the aggregation of oil molecules. According to Chen et al. [20], these conditions cause a reduction in the oil–water interaction to form an emulsion. The EAI is affected by the solubility, conformation and surface properties of protein. Due to variations in the solubility between surfaces, most proteins are less soluble at their pI and have a low emulsifying capacity [58]. Apart from various sources of raw materials, the pH level was found to be able to alter the ability of the obtained emulsions due to the effects of their amphoteric and hydrophobic properties on the peptide chains.

## 4. Conclusions

Collagens from the skin of barracuda (*Sphyraena* sp.) were extracted using three acid solutions (i.e., acetic, lactic and citric acids). Lactic acid-solubilized collagen (LSBS) had the highest yield (*p* < 0.05) compared to citric acid- and acetic acid-solubilized collagens (CSBS and ASBS). However, the ASBS sample had superior characteristics, particularly in terms of its thermal stability, solubility and color attributes. In terms of its thermostability, a higher *T_max_* value observed in ASBS was in line with the higher Hyp content as aforementioned. Other properties tested by, for example, XRD and FTIR analysis, resulted in the triple helical structures of the acid-solubilized collagens being maintained, indicating that all the collagens were undenatured during solubilization with acid solution. Taken together, the ASBS sample is more favorable based on its properties and it could be used as an alternative collagen.

## Figures and Tables

**Table 1:** The yields, Hyp content and color parameters of acid-solubilized collagens from barracuda skin.

Sample	Yield (g/100 g)	Hyp (mg/g)	Color Parameters		
			L*	a*	b*
ASBS	6.77 ± 0.10 ^c^	82.78 ± 0.19 ^a^	78.54 ± 4.67 ^a^	−0.05 ± 0.06 ^b^	0.64 ± 0.56 ^b^
LSBS	10.06 ± 0.50 ^a^	81.76 ± 0.05 ^b^	56.88 ± 4.29 ^b^	0.60 ± 0.16 ^a^	5.83 ± 1.45 ^a^
CSBS	8.53 ± 0.60 ^b^	81.97 ± 0.14 ^b^	54.34 ± 1.95 ^b^	0.66 ± 0.24 ^a^	3.78 ± 1.67 ^a^

All data are presented as the mean ± standard deviation (SD, *n* = 3). The different superscript letters in the same column are significantly different (*p* < 0.05). ASBS: acetic-solubilized barracuda skin collagen; LSBS: lactic-solubilized barracuda skin collagen; CSBS: citric-solubilized barracuda skin collagen; Hyp: hydroxyproline; L*: lightness; a*: redness (green to red); b*: yellowness (blue to yellow).

**Table 2:** The peak areas and descriptions of acid-solubilized collagens.

Peak Area (cm^−1^)			Peak Description
ASBS	LSBS	CSBS	
3278.28	3277.35	3282.94	Amide A: N–H stretch, coupled with hydrogen bond
2921.38	2920.44	2921.38	Amide B: CH_2_ symmetric and asymmetric stretch
1628.89	1628.89	1628.89	Amide I: C=O stretch, coupled with hydrogen bond
1541.29	1541.29	1541.29	Amide II: N–H bend coupled with C–N stretch
1233.78	1236.58	1234.71	Amide III: CH_2_ wagging of proline

ASBS: acetic-solubilized barracuda skin collagen; LSBS: lactic-solubilized barracuda skin collagen; CSBS: citric-solubilized barracuda skin collagen.

**Table 3:** XRD and DCS analyses of acid-solubilized collagens from barracuda skin.

Sample	XRD Test				DSC Profile	
	Peak 1 (Sharp Peak)		Peak 2 (Broad Peak)			
	2*θ*	d (nm)	2*θ*	d (nm)	*T_max_* (°C)	Δ*H* (J/g)
ASBS	7.48	1.18	20.02	0.44	41.29	0.13
LSBS	7.26	1.22	19.16	0.46	40.69	0.08
CSBS	7.64	1.16	19.12	0.46	40.16	0.05

ASBS: acetic acid-solubilized barracuda skin collagen; LSBS: lactic acid-solubilized barracuda skin collagen; CSBS: citric acid-solubilized barracuda skin collagen.

**Table 4:** WAC and OAC values of acid-solubilized collagens from barracuda skin.

Sample	ASBS	LSBS	CSBS
WAC (mL/g)	18.50 ± 1.50 ^a^	16.50 ± 0.50 ^a^	13.00 ± 1.40 ^a^
OAC (mL/g)	13.00 ± 0.10 ^a^	6.00 ± 1.00 ^b^	11.00 ± 0.00 ^a^
EAI (pH 4.0) (m^2^/g)	206.69 ± 2.19 ^a^	320.12 ± 4.62 ^a^	289.82 ± 3.76 ^a^
EAI (pH 7.0) (m^2^/g)	145.48 ± 2.01 ^b^	76.46 ± 1.58 ^b^	68.77 ± 1.42 ^b^
EAI (pH 10.0) (m^2^/g)	78.57 ± 1.90 ^c^	49.44 ± 3.98 ^c^	37.62 ± 2.62 ^c^

All data are presented as the mean ± standard deviation (SD, *n* = 3). The different lowercase letters in the same column are significantly different (*p* < 0.05). ASBS: acetic-solubilized barracuda skin collagen; LSBS: lactic-solubilized barracuda skin collagen; CSBS: citric-solubilized barracuda skin collagen.

## Data Availability

The data presented in this study are available upon request from the corresponding author.
